# Complete plastome sequence of *Hoya pottsii* Traill and *Hoya liangii* Tsiang (Apocynaceae)

**DOI:** 10.1080/23802359.2018.1524720

**Published:** 2018-10-26

**Authors:** Xin-Hang Tan, Jian-Hua Wang, Kun-Kun Zhao, Zhi-Xin Zhu, Hua-Feng Wang

**Affiliations:** Hainan Key Laboratory for Sustainable Utilization of Tropical Bioresources, Institute of Tropical Agriculture and Forestry Hainan University, Haikou, China

**Keywords:** *Hoya pottsii* Traill, *Hoya liangii* Tsiang, plastome, phylogeny, genome structure, Asclepiadoideae, Apocynaceae

## Abstract

*Hoya* is the largest genus (about 350–450 species) within Apocynaceae. It is a subshrub or liana, epiphytic or epilithic. Most species grow in tropical and subtropical South and Southeast Asia. Here we report and characterize the complete plastid genome sequence of *Hoya pottsii* Traill *and Hoya liangii* Tsiang in an effort to provide genomic resources useful for promoting its systematics research. The complete plastome of *H. pottsii* is 161,565 bp in length, including two Inverted Repeat (IR) regions of 24,657 bp, a Large Single-Copy (LSC) region of 92,532 bp, and a Small Single-Copy (SSC) region of 19,719 bp. The plastome contains 115 genes, consisting of 81 unique protein-coding genes, 30 unique tRNA genes, and 4 unique rRNA genes. The overall A/T content in the plastome of *H. pottsii* is 62.40%. The complete plastome of *H. liangii* is 162,989 bp in length, including two IR regions of 24,841 bp, a LSC region of 93,292 bp, and a SSC region of 20,015 bp. The plastome contains 115 genes, consisting of 81 unique protein-coding genes, 30 unique tRNA genes, and 4 unique rRNA genes. The overall A/T content in the plastome of *H. pottsii* is 62.30%. The complete plastome sequence of *H. pottsii* and *H. liangii* will provide a useful resource for the conservation genetics of the two species as well as for the phylogenetic studies of *Hoya*.

## Introduction

*Hoya* is the largest genus (about 350–450 species) within Apocynaceae. It is a subshrub or liana, epiphytic or epilithic. Most species grow in tropical and subtropical South and Southeast Asia (Lamb and Rodda 2016). There are 38 species and one variety in China, and most of them are distributed in southwestern China (Zhang et al. 2015). The leaves of *H. pottsii* are used for the treatment of fractures and swellings and for draining off pus and promoting new growth. Consequently, the genetic and genomic information is urgently needed to promote its medicinal and systematics research of *H. pottsii* and *H. liangii*. Here, we report and characterize the complete plastome of *H. liangii* (GenBank accession number, MH678666, this study) and *H. pottsii* (MH678667, this study). This is the first report of a complete plastome for the genus *Hoya*.

In this study, *H. pottsii* and *H. liangii* were sampled from Luoyi village, Chengmai county in Hainan province of China (110.111°E, 19.897°N). The voucher specimens (*H. pottsii*, Wang et al., B265 and *H.liangii*, Wang et al., B266) were deposited in the Herbarium of the Institute of Tropical Agriculture and Forestry (HUTB), Hainan University, Haikou, China.

The experiment procedure is as reported in Zhu et al. (2018). Around 6 Gb clean data were assembled against the plastome of *Cynanchum wilfordii* (KT220733.1) (Jang et al. 2016) using MITObim v1.8 (Hahn et al. 2013). The plastome was annotated using Geneious R8.0.2 (Biomatters Ltd., Auckland, New Zealand) against the plastome of *C. wilfordii* (KT220733.1). The annotation was corrected with DOGMA (Wyman et al. 2004).

The plastomes of *H. pottsii* and *H. liangii* were found to possess a total length 161,565 bp and 162,989 bp, respectively. They both have typical quadripartite structure of angiosperms, containing two Inverted Repeats (IRs) of 24,657 bp in *H. pottsii* and 24,841 bp in *H. liangii*, a Large Single-Copy (LSC) region of 92,532 bp (*H. pottsii*) and 93,292 bp (*H. liangii*), a Small Single-Copy (SSC) region of 19,719 bp (*H. pottsii*) and 20,015 bp (*H. liangii*). The plastome of *H. pottsii* and *H. liangii* contains 115 genes, consisting of 81 unique protein-coding genes (7 of which are duplicated in the IR), 30 unique tRNA genes (7 of which are duplicated in the IR), and 4 unique rRNA genes. Among these genes, 5 pseudogenes (*rpoC1*, *accD*, *ycf2*, *ndhH*, *ycf1*) in *H. pottsii* and 6 pseudogenes (*rpoC2*, *accD, ycf2*, *ycf1*, *ndhH*, *ycf1*) in *H. liangii*, 14 genes possessed a single intron and 3 genes (*ycf3, clpP, rps12*) had 2 introns. The gene *rps12* was found to be trans-spliced, as is typical of angiosperms. The overall A/T content in the plastome of *H. pottsii* is 62.40%, which the corresponding value of the LSC, SSC, and IR region were 64.10%, 68.20%, and 56.80%, respectively. The overall A/T content in the plastome of *H. liangii* is 62.30%, which the corresponding value of the LSC, SSC, and IR region were 64.10%, 68.20%, and 56.80%, respectively.

**Figure 1.**
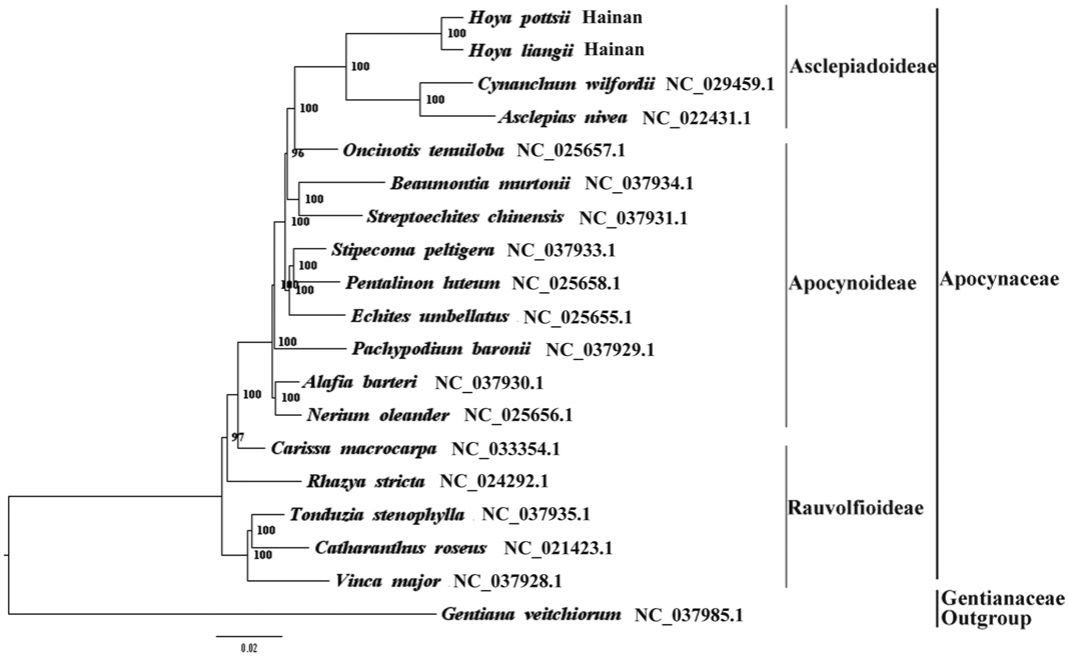
The best ML phylogeny recovered from 19 complete plastome sequences by RAxML. Accession numbers: *Hoya pottsii* Traill (MH678667, this study), *Hoya liangii* Tsiang (MH678666, this study), *Cynanchum wilfordii* NC_029459.1, *Asclepias nivea* NC_022431.1, *Oncinotis tenuiloba* NC_025657.1, *Beaumontia murtonii* NC_037934.1, *Streptoechites chinensis* NC_037931.1, *Stripecoma peltigera* NC_037933.1, *Pentalinon luteum* NC_025658.1, *Echites umbellatus* NC_025655.1, *Pachypodium baronii* NC_037929.1, *Alafia barteri* NC_037930.1, *Nerium oleander* NC_025656.1, *Carissa macrocarpa* NC_033354.1, *Rhazya stricta* NC_024292.1, *Tonduzia stenophylla* NC_037935.1, *Catharanthus roseus* NC_021423.1, *Vinca major* NC_037928.1; outgroup: *Gentiana veitchiorum* NC_037985.1.

We used RAxML(Stamatakis 2006) with 1000 bootstraps under the GTRGAMMAI substitution model to reconstruct a maximum likelihood (ML) phylogeny of 16 published complete plastome of Apocynaceae, using *Gentiana veitchiorum* (Gentianaceae) as outgroup. The phylogenetic analysis indicated that *H. pottsii* and *H. liangii* are close to *C. wilfordii* and *Asclepias nivea* within Apocynaceae respectively (Figure 1). Most nodes in the plastome ML trees were strongly supported. The complete plastome sequence of *H. pottsii* and *H. liangii* will provide a useful resource for the conservation genetics of the two species as well as for the phylogenetic studies of *Hoya*.
